# Transplantable Subcutaneous Hepatoma 22a Affects Functional Activity of Resident Tissue Macrophages in Periphery

**DOI:** 10.1155/2011/793034

**Published:** 2011-07-03

**Authors:** Ekaterina P. Kisseleva, Andrei V. Krylov, Olga I. Stepanova, Victoria I. Lioudyno

**Affiliations:** Institute for Experimental Medicine, Acad. Pavlov Stree 12, Street Petersburg 197376, Russia

## Abstract

Tumors spontaneously develop central necroses due to inadequate blood supply. Recent data indicate that dead cells and their products are immunogenic to the host. We hypothesized that macrophage tumor-dependent reactions can be mediated differentially by factors released from live or dead tumor cells. In this study, functional activity of resident peritoneal macrophages was investigated in parallel with tumor morphology during the growth of syngeneic nonimmunogenic hepatoma 22a. Morphometrical analysis of tumor necroses, mitoses and leukocyte infiltration was performed in histological sections. We found that inflammatory potential of peritoneal macrophages in tumor-bearing mice significantly varied depending on the stage of tumor growth and exhibited two peaks of activation as assessed by nitroxide and superoxide anion production, 5′-nucleotidase activity and pinocytosis. Increased inflammatory reactions were not followed by the enhancement of angiogenic potential as assessed by Vascular Endothelial Growth Factor mRNA expression. Phases of macrophage activity corresponded to the stages of tumor growth characterized by high proliferative potential. The appearance and further development of necrotic tissue inside the tumor did not coincide with changes in macrophage behavior and therefore indirectly indicated that activation of macrophages was a reaction mostly to the signals produced by live tumor cells.

## 1. Introduction

It is now abundantly evident that innate immune response plays important role in antitumor defense [1]. One particular inflammatory cell type, the macrophage, has emerged as a central regulator of tumor onset and progression. 

Macrophages represent blood-borne-derived descendants of mononuclear cells which migrate from the circulation into tissues and display a high degree of plasticity, which is tuned by the tissue microenvironments where they reside. It was shown that macrophages play a dual role in tumor growth and can possess antitumor as well as protumor activities. Therefore they were subdivided into M1 (classically activated) or M2 (alternatively activated) phenotypes [2]. 

In order to be fully activated macrophages need to be exposed to two signals: IFN-*γ* and microbial product LPS. After that macrophages exhibit a proinflammatory M1 phenotype which is characterized by the release of proinflammatory cytokines (IL-12, TNF-*α*), reactive nitrogen intermediates (NOs), and reactive oxygen intermediates (ROIs), which support their microbicidal/tumoricidal activity. M1 macrophages promote Th1 responses and are considered to be powerful effectors against pathogens and tumor cells. 

On the contrary, M2 cell phenotype is induced in response to Th2 cytokines IL-4, IL-13, and IL-10, as well as by apoptotic cells and immune complexes. Several data indicate that under the influence of tumor environment macrophages exhibit predominantly M2 phenotype which is characterized by increased production of arginase-1 and reduced NO production, enhanced release of anti-inflammatory cytokines (IL-10), ability to support angiogenesis, and tissue remodeling [3]. Additionally, M2 macrophages are less efficient in presenting antigens as well as eliciting antitumor T cell responses and can promote tumor growth. 

However, several other data have indicated that tumor-associated macrophages express both M1- and M2-like characteristics thus demonstrating a “mixed” phenotype [4, 5]. Peripheral macrophages were also shown to possess features of neither M1 nor M2 phenotype: had downregulated NF-*κ*B-dependent proinflammatory signaling pathways and IL-12 production, as well as no markers typical to M2 macrophages, such as enhanced expression of IL-10 or arginase [6]. 

These data indicate that M1/M2 classification needs to be specified. Moreover, it is now well accepted that there exist at least four different types of adaptive immune responses, namely, Th1, Th2, Th17, and T-regulatory cells type; and innate immune reactions provide a special microenvironment for each of them [7]. Therefore it is suggested to be at least four different types of innate immune responses. 

The contribution of macrophages from extratumor sites to the host immunity has been investigated in a very few papers and much less is known on their proangiogenic functions. Several tumor-derived factors are known to stimulate production of angiogenic factors by macrophages. For example, TNF-*α* secreted by ovarian tumor cell line in vitro enhances the release of Vascular Endothelial Growth Factor (VEGF) by human macrophages during cocultivation [8]. Peritoneal macrophages isolated from mice bearing melanoma B16F10 produced significant amounts of VEGF [9].

Finally, there is now a growing interest to the data indicating that dead cells and their products are immunogenic to the host and can activate innate immune response [10]. Besides, it is well established that tumors spontaneously develop central necrosis due to inadequate and heterogeneous vascular network that leads to insufficient blood supply [11]. Therefore, we hypothesized that macrophage tumor-dependent reactions can be mediated differentially by factors released from live or dead tumor cells.

In this study, resident peritoneal macrophages were investigated in parallel with tumor morphology during the growth of syngeneic nonimmunogenic hepatoma 22a. Several parameters attributed to inflammatory and angiogenic macrophage potential were studied: the production of cytotoxic factors such as NO and ROI [12], 5′-nucleotidase (5′-N) activity (CD73) as a marker for purine metabolism, and pinocytosis to characterize macrophage endocytic activity. Additionally, ability of macrophages to synthesize proangiogenic (VEGF) and anti-angiogenic (thrombospondin-1, TSP-1) [13] factors was investigated. 

We found that inflammatory potential of peritoneal macrophages in tumor-bearing mice significantly varied depending on the stage of tumor growth and exhibited two peaks of activation as assessed by abovementioned parameters. These phases corresponded to increased activity of the tumor growth characterized by high proliferative potential. The appearance and further development of necrotic tissue inside the tumor did not coincide with changes in macrophage behaviour and therefore indirectly indicated that activation of macrophages was a reaction mostly to the signals produced by live tumor cells. 

## 2. Material and Method

### 2.1. Animals

C3HA male mice weighing 18–20 g, three months old, were purchased from “Rappolovo” Animal Farm, Russian Academy of Medical Sciences, St. Petersburg, Russia. All experiments were performed using protocols approved by the Russian Animal Ethics Committee and followed institutional animal use and care guidelines.

### 2.2. Tumor

The cell line of hepatoma 22a (MH-22a) was obtained from tissue culture collection of Institute of Cytology, Russian Academy of Sciences, St. Petersburg, and was passaged in vitro in DMEM supplemented with 10% fetal calf serum (Sigma). Cells originated from the solid nonmetastasizing murine tumor induced by 3-methylcholantrene in C3HA mice [14] and were further adapted for in vitro growth [15].

### 2.3. Transplantation Test

In our studies hepatoma 22a was characterized as a nonimmunogenic tumor by transplantation test. Ten days after i.p. immunization with 10^7^ lethally irradiated (100 Gy) cells, mice were s.c. inoculated with 10^3^–10^6^ live hepatoma cells, five animals per group. During 4 weeks the dynamics of tumor growth in previously immunized animals was compared to control mice. Tumor volume was determined by using the formula 0.4 × ab^2^, where “*a*” and “*b*” stood for the largest and the smallest tumor size, respectively [16]. As shown in Table 1, vaccination was non-effective and did not prevent or retard tumor growth.

### 2.4. Subcutaneous Tumor Model

In all further experiments we used only live hepatoma cells for the injections. For the development of solid tumors 10^5^ live hepatoma cells in 0.5 mL PBS were inoculated s.c. on the back. Control mice received injection of PBS only. Mice were sacrificed by cervical dislocation on 3, 7, 14, 21, 28, and 35 day after starting experiment. 

### 2.5. Macrophage Cultures

Resident peritoneal macrophages were obtained by peritoneal lavage with injecting 5 mL of PBS and pooled from 5 mice in each group. Peritoneal cells were left for 2 hours to adhere to the bottom of 96-well plates and nonadherent cells were discarded. Macrophages were cultured in RPMI 1640 media supplemented with 10% FCS and kept in incubator at 37°C, 5% CO_2_ during 24 hours. Nitrite production was measured as spontaneous or induced by adding 10 ng/mL LPS (E. Coli B5-055, Sigma) [17]. Superoxide production (nitroblue tetrazolium test, NBT-test) was measured as spontaneous or induced by adding 1 *μ*g/mL 12-O-tetradecanoylphorbol-13-acetate (TPA, Sigma) [18]. Fluid-phase pinocytosis of neutral red dye was studied as published earlier [19]. 5′-nucleotidase (5′-N) activity was measured by colorimetric estimation of inorganic phosphate released from AMP as described previously [20]. 

### 2.6. Cellular Composition of Peritoneal Exudate Cells (PECs) and Peripheral Blood Leukocytes

It was determined in Giemsa-stained smears. Blood was collected by retroorbital puncture and the number of nucleated cells was counted with the help of hemocytometer.

### 2.7. Thymi and Spleens

They were isolated and weighed and number of nucleated cells per organ counted.

### 2.8. Reverse-Transcription PCR Assay

The level of VEGF, VEGFR-1, VEGFR-2, VEGFR-3, and TSP-1 mRNA expression was studied by semiquantitative reverse transcription followed by polymerase chain reaction (RT-PCR). Total RNA was isolated from macrophages by a single-step method using TRI reagent (Sigma) from each mouse individually. In some experiments thymocytes were also studied. Synthesis of cDNA templates was carried out using 2 *μ*g of total RNA, olygo(dT) 15 primers, and M-MLV reverse transcriptase (Promega) according to the manufacturer's instructions. Specific primers used for PCR amplifications were designed spanning an intron so that the size of reaction product from cDNA and nuclear DNA was different. The primers for VEGF were sense 5′-gaccctggctttactgctgta-3′ and antisense 5′-gtgaggtttgatccgcatgat-3′ (product size, 297 bp), being common to all three known murine VEGF isoforms. Primers for VEGFR-1 were sense 5′-gaagcggttcacctggactgagacc-3′ and antisense 5′-ggctttgctggggggatttctctaa-3′ (product size 432 bp); for VEGFR-2: sense 5′-acagacagtgggatggtccttgcat-3′ and antisense 5′-aaacaggaggtgagctgcagtgtgg-3′ (272 bp); for TSP-1: sense 5′-caaaggagatgcctgtgacc-3′ and antisense 5′-ctggaatcgtcggaaatcgg-3′ (product size 216 bp). As a control for housekeeping gene an RT-PCR procedure for *β*-actin was also delivered. Primers for *β*-actin were sense 5′-atggatgacgatatcgct-3′ and antisense 5′-atgaggtagtctgtcaggt-3′ (568 bp). PCR was carried out in a thermal cycler (Techne) using initiation of denaturation at 94°C for 5 minutes and cycles of denaturation at 94°C for 1 min, annealing at 59°C for 1 min, and extension at 72°C for 1 min (repeated for 30 cycles), with a final end extension of 5 min at 72°C for all targets tested. PCR products were visualized by electrophoresis through 1, 5% agarose gels, stained with ethidium bromide, photographed and analyzed by using image analysis software (Scion Image).

### 2.9. Serum Cytokine Level Detection

At different time points when mice were sacrified they were bled to collect peripheral blood. Then, sera were obtained by centrifugation and stored at −20°C until being analyzed. Serum levels of TNF-*α*, IL-6, and VEGF were studied from each mouse individually in duplicate with appropriate ELISA kits for mouse cytokines (“Genzyme”, “R&D Systems” and “Bender Medsystems”, USA). 

### 2.10. Histology

Excised tumor nodules were fixed in 10% formalin and embedded in paraffin. Microscopic sections (5 *μ*m) were stained with azure-II-eosin and hematoxylin-eosin. The alignment of the areas occupied in sections by necrotic and viable tumor tissue was calculated by using Weibel screen with 50 test points at magnification ×200 in 10 fields of vision [21]. The number of macrophages, lymphoid, and mast cells infiltrating peripheral areas of the tumor was calculated at magnification of ×600 per 10 fields of vision and the number of mitoses in viable areas of the tumors at magnification ×900 per 10 fields of vision with calculation of the average out for each animal studied.

### 2.11. Statistics

Data were analyzed using Student's *t*-test (*P* < .05).

## 3. Results

### 3.1. Tumor Growth Affects Inflammatory Properties of Macrophages

To evaluate the inflammatory phenotype of peritoneal macrophages we studied six different parameters: spontaneous and LPS-induced NO_2_-production, spontaneous and TPA-induced superoxide anion production (NBT-test), pinocytosis, and 5′-N activity.

**Figure 1:**
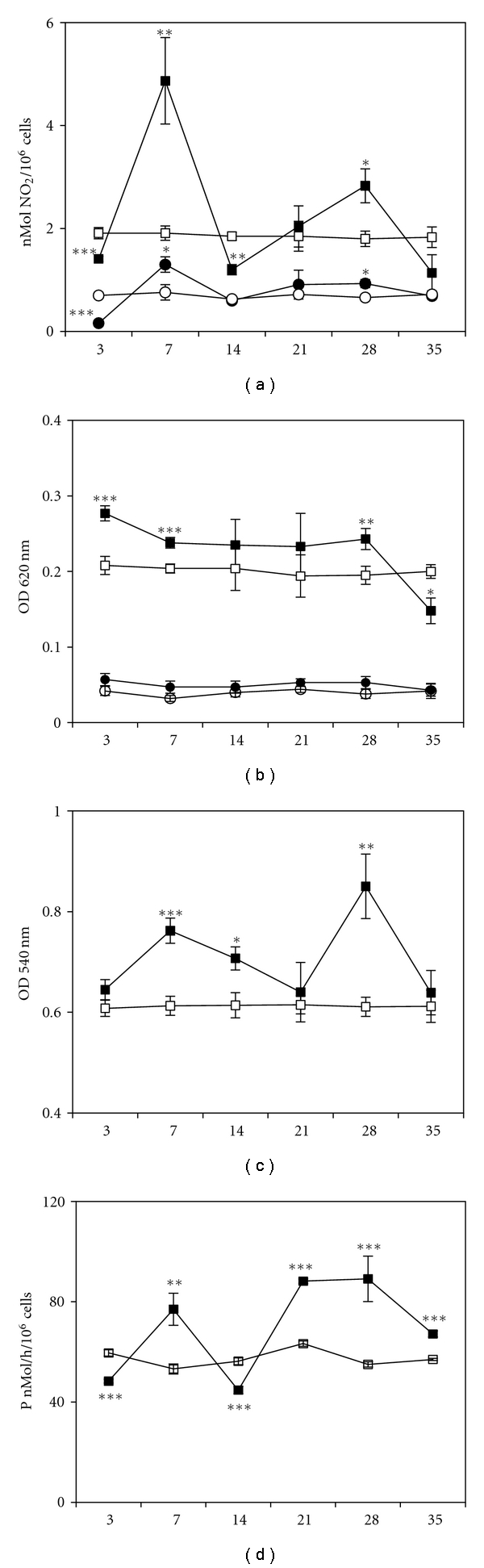
Dynamics of functional activity of peritoneal macrophages from mice bearing hepatoma 22a. *X*-axis: time after inoculation of the tumor, days. *Y*-axis: (a) Spontaneous NO_2_-production by macrophages from ○—control mice and *⚫*—tumor-bearers; NO_2_-production in macrophages stimulated with 10 ng/mL LPS during 24 h, nM/h/10^6^ cells: □—in control mice and ■—in tumor-bearers. (b) Spontaneous NBT-test: ○—in macrophages from control mice, *⚫*—from tumor-bearers; NBT-test in macrophages stimulated with 1 *μ*g/mL TPA during 1 h: □—in control mice, ■—in tumor-bearers. Optical density was measured at *λ* = 620 nm. (c) Fluid-phase pinocytosis of neutral red during 1 h, optical density was measured at *λ* = 540 nm; □—macrophages from control mice, ■—from tumor-bearers. (d) 5′-nucleotidase activity was measured by colorimetric estimation of inorganic phosphate released from AMP at *λ* = 690 nm, *P* nM/h/10^6^ cells; □—macrophages from control mice, ■—from tumor-bearers. Each point represents the results of 4 independent experiments tested in quadriplicates (M ± SEM). Control mice received PBS. *P < .05, **P < .01, ***P < .001 compared to control animals.

NO_2_-production fluctuated up and down with two points of decrease (3 and 14 days) and two points of increase (7 and 28 days). The difference between stimulated and spontaneous production on 7 day of tumor growth was 3 times more than in control mice, and on day 28 was about 1.5 times more than in non-tumor bearers (Figure 1(a)). TPA-induced superoxide anion production was upregulated on day 3, 7, and 28 and downregulated at the late stage of tumor growth—35 day (Figure 1(b)). Fluid-phase pinocytosis was increased at 3, 7, and 28 days and showed no decrease (Figure 1(c)). Taken together, the results of three tests revealed two evident peaks of activity at 7 and 28 days. 

The most specific changes were found in the activity of membrane enzyme 5′-N in macrophages. This was the only one characteristic that altered at all time-points of the study. This parameter showed significant fluctuations and was downregulated at 3 and 14 days and upregulated at 7, 21, 28 and 35 days (Figure 1(d)). The decrease of 5′-N activity in macrophages is usually considered to be a sign of cell activation [22], whereas increased activity may be interpreted differently [23, 24].

We next examined how these inflammatory changes corresponded to macrophage mRNA synthesis of angiogenic or anti-angiogenic factors.

### 3.2. The Influence of Tumor Growth on the Expression of Angiogenic Phenotype in Macrophages

**Figure 2:**
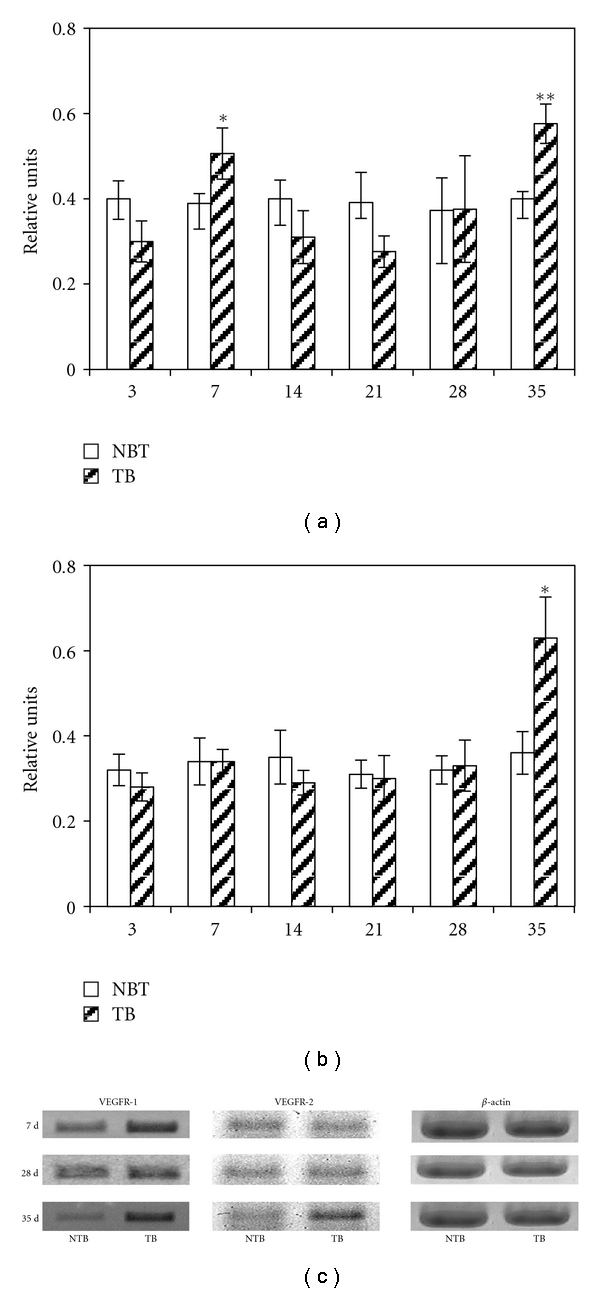
Hepatoma growth increases VEGFR-1 (a) and VEGFR-2 mRNA expression (b) in peritoneal macrophages. Total RNA was isolated from peritoneal cells from each mouse individually. Two *μ*g of total RNA were used to perform semiquantitative (reverse-transcription) RT-PCR as described; mRNA levels specific for VEGFR-1 and VEGFR-2 were normalized to *β*-actin. Each bar represents the average obtained from 8 mice (M ± SEM); *P < .05, **P < .01 compared to control. (c) Representative RT-PCR analysis for VEGFR-1 and VEGFR-2 mRNA expression in macrophages. NTB: nontumor bearing mice; TB: mice with hepatoma 22a.

We expected that inflammatory changes of macrophage activity would be accompanied by certain alterations in their angiogenic properties. Therefore we studied mRNA expression of main angiogenic factor—VEGF and its receptors, as well as anti-angiogenic factor—TSP-1. Unexpectedly, we did not found any difference in VEGF mRNA expression in peritoneal macrophages of tumor-bearing mice at any time of the study (3, 7, 14, 21, 28, or 35 days) as compared to control animals (data not shown). There was slight enhancement of VEGFR-1 mRNA expression at 7 and 35 days of tumor growth and VEGFR-2 mRNA increase at 35 day (Figure 2). 

**Figure 3:**
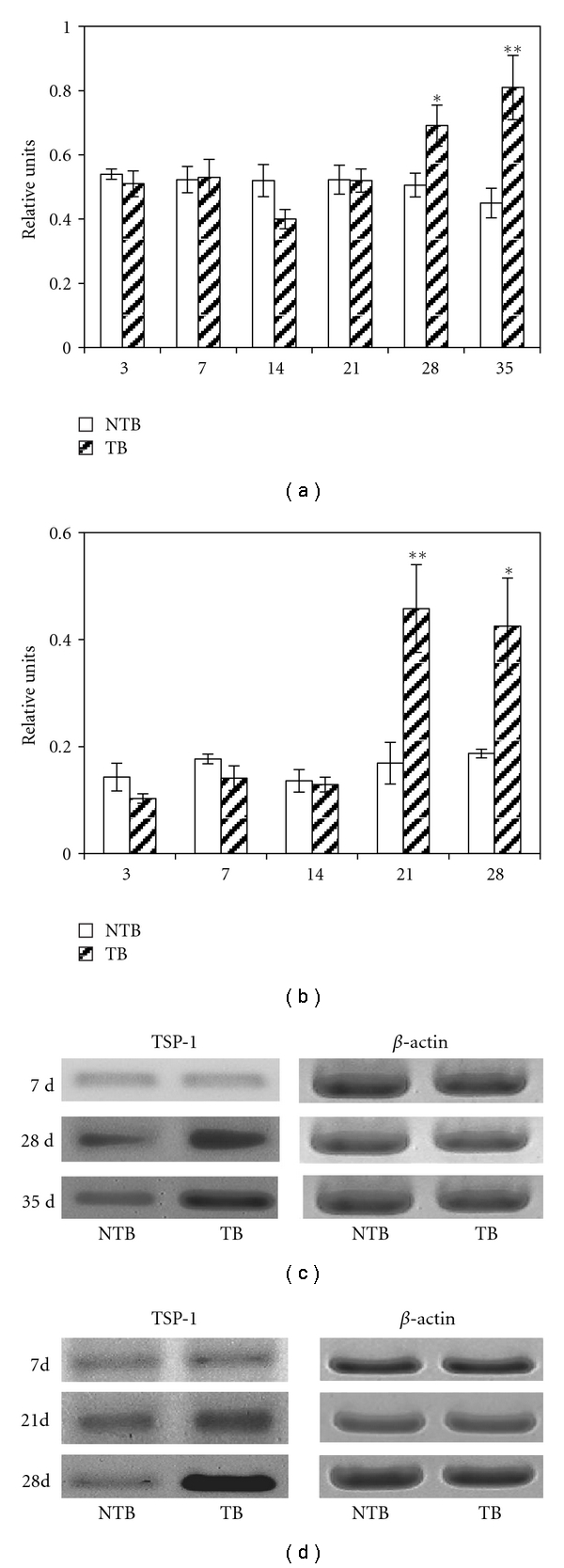
Hepatoma growth increases TSP-1 mRNA expression in peritoneal macrophages (a) and thymocytes (b). Total RNA was isolated from peritoneal cells or thymocytes from each mouse individually. Two *μ*g of total RNA were used to perform semiquantitative (reverse-transcription) RT-PCR as described; mRNA levels specific for TSP-1 were normalized to *β*-actin. Each bar represents the average obtained from 8 mice (M ± SEM); *P < .05, **P < .01 compared to control. (c) Representative RT-PCR analysis for TSP-1 mRNA expression in macrophages. (d) Representative RT-PCR analysis for TSP-1 mRNA expression in thymocytes. Thymocytes on the 35 day were not studied because of the thymic involution. NTB: nontumor bearing mice; TB: mice with hepatoma 22a.

Opposite to VEGF, TSP-1 mRNA expression in macrophages was shown to increase at the late stages of tumor growth (Figures 3(a) and 3(c)). To examine whether such increase was systemic in tumor-bearing mice we also measured TSP-1 mRNA expression in the thymus, which was another organ distant to the tumor site. We found that at the 28 days of tumor growth TSP-1 mRNA expression in thymocytes was similarly enhanced (Figures 3(b) and 3(d)). We did not study thymocytes at the 35 day because thymi were significantly depleted at this time. 

### 3.3. Morphological Characteristics of Tumor Growth

**Figure 4:**
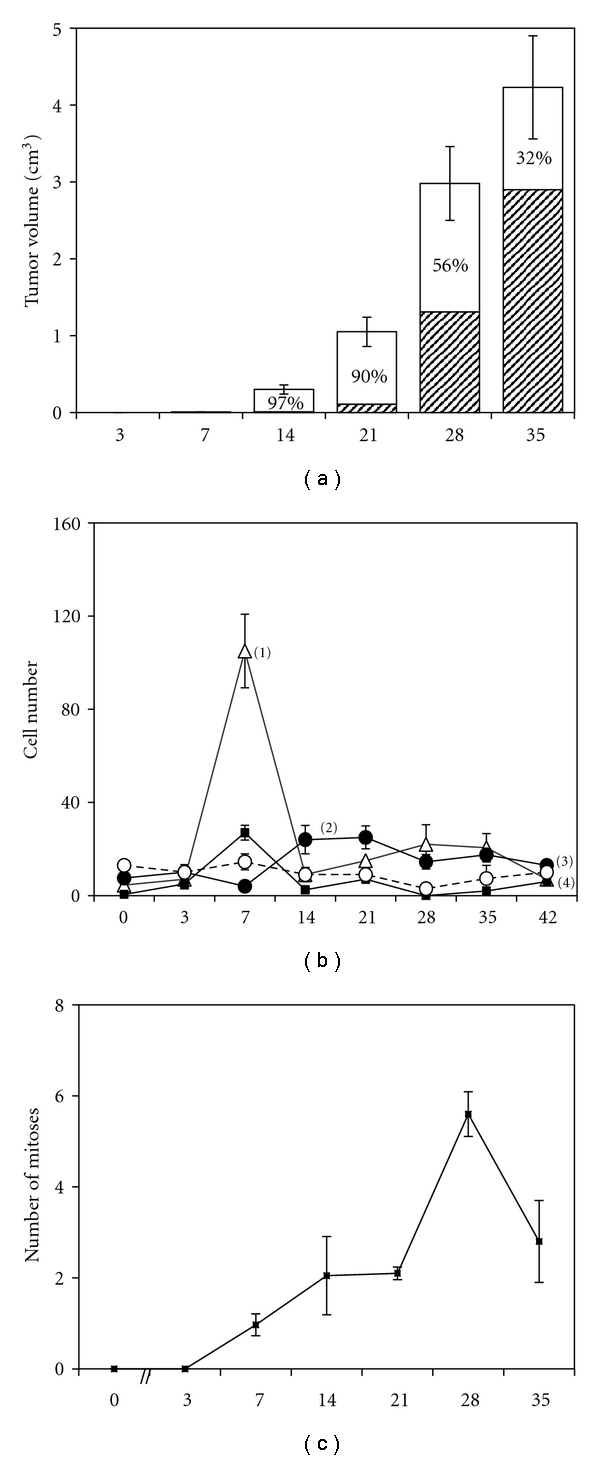
Morphological and histological parameters of hepatoma 22a growth (a–c). (a) Tumor volume after s.c. inoculation of 10^5^ live hepatoma cells was measured on days of sacrifice; bars represent areas of viable (empty parts of the bar chart) and necrotized (dashed parts of the bar chart) tumor tissue in percent to the whole tumor volume. Areas of viable and necrotic tissues were calculated in histological sections per field of vision, magnification ×200; ten fields of vision were counted for each mice (*n* = 3) (b) The mean number of lymphocytes (1), mast cells (2), polymorphonucleus leukocytes (3), and macrophages (4), infiltrating periphery of the tumor (one field of vision from the edge of the tumor), magnification of ×600 per 10 fields of vision (*n* = 3) (c) The mean number of mitoses in viable areas of the tumors at magnification ×900 per 10 fields of vision (*n* = 3). All values are the mean ± SEM.

Tumors remained invisible several days after inoculation. At 7 days tumors represented a small palpable nodules about 2-3 mm in diameter. The tumor grew fast within 3 weeks and reached a steady state after 28 day (Figure 4(a)). 

**Figure 5:**
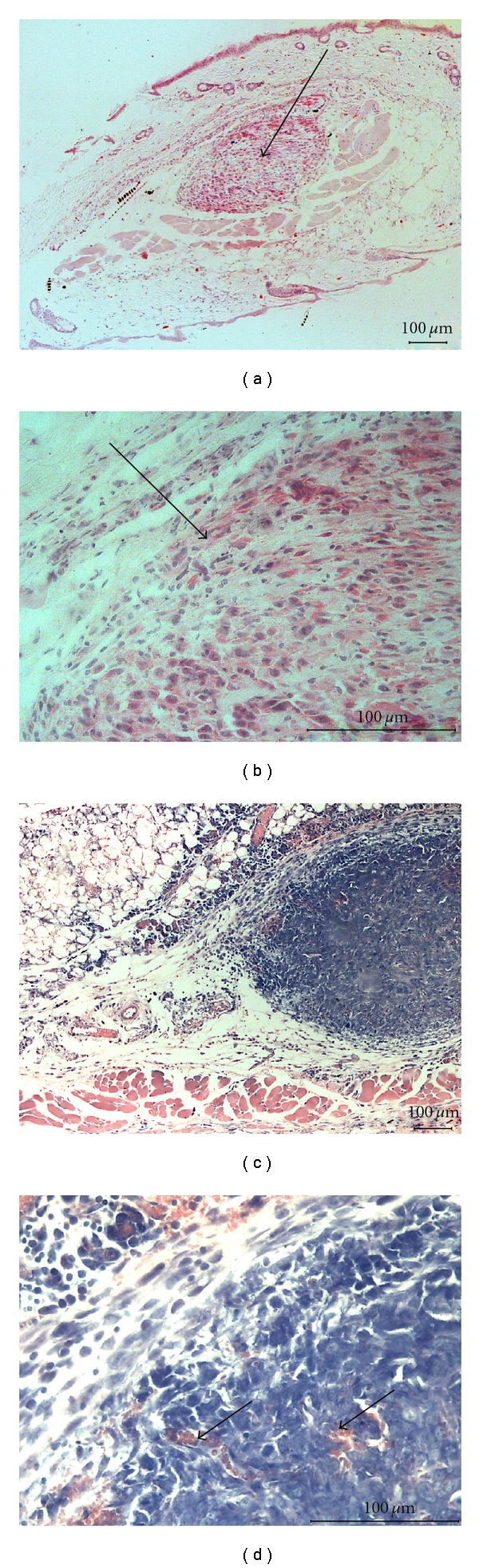
Microphotographs of hepatoma 22a (a) Tumor at 3 day after inoculation ×200 (b) higher magnification ×600: Polymorphous, separately lying tumor cells, no reaction from peripheral tissue. Tumor tissue is shown by arrows. Hematoxylin-eosin staining (c) tumor at 7 day after inoculation ×200 (d) higher magnification ×600. The appearance of new vessels in the tumor tissue shown by arrows. (Azure-II-eosin staining).

Histological study revealed that 3 days after inoculation tumor cells were located separately, isolated from each other, and were polymorphous, without mitoses or perifocal infiltration (Figures 5(a) and 5(b)). Single polymorphonucleus leukocytes were visible around the tumor. 

**Figure 6:**
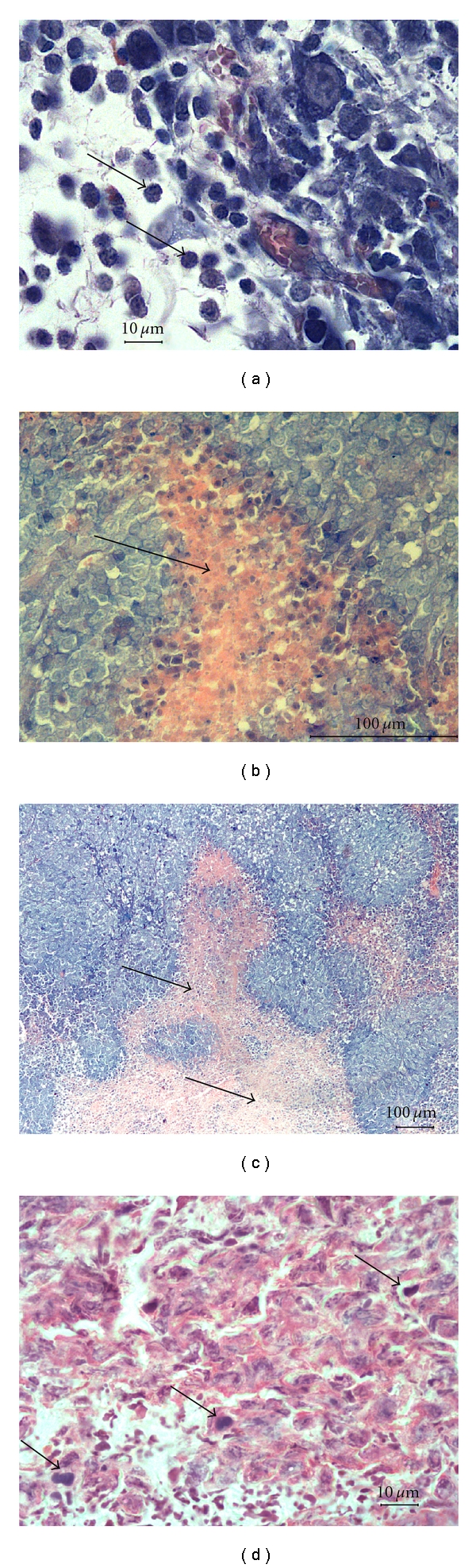
Microphotographs of hepatoma 22a. (a) Tumor at 7 day after tumor inoculation ×900. Mononuclear infiltration at the periphery of tumor nodule is shown by arrows. Azure-II-eosin staining. (b) Tumor at 14 day after inoculation ×600. The appearance of first central necrosis is shown by arrow. Azure-II-eosin staining. (c) Tumor at 21 day after inoculation ×200. Necrotic tissue (shown by arrows). Azure-II-eosin staining. (d) Tumor at 28 day after inoculation ×900; Mitosis in the remaining viable tumor tissue. Hematoxylin-eosin staining.

At the 7th day, the tumor tissue was more compact, organized, and started to be vascularized from periphery (Figures 5(c) and 5(d)). Simultaneously with new capillaries we observed at the periphery of tumor nodule the appearance of mononuclear infiltration, here and there accumulation of 10–15 lymphocytes per field of vision (Figures 4(b) and 6(a)). Later on, cellular infiltration significantly diminished except for the mast cells (2–5 cells per field of vision at 14–28 day, Figure 4(b)).

At 14 days tumor diameter was about 10 mm. Examination of sections showed that tumor cells became more homogeneous, moderately differentiated, and typical for trabecular hepatoma. Mononulear cells at the periphery of the tumor nodule were almost absent. In the central parts adjacent to necrotic areas there were visible foci of white blood cell infiltration (Figure 6(b)). Starting from this point we observed the development of central necrosis, the surface area of which grew constantly and reached by day 35 about 70 per cent of the section area. When we calculated these percentages proportional to the tumor volume, we found out that the volume and the rate of growth of viable tumor tissue were the largest at 28 day and after that significantly diminished at 35 day (Figure 4(a), Table 2). 

At the late stages of tumor growth the necrotized areas were almost homogenous with no live tumor or infiltrating cells evident (Figure 6(c)). Viable tumor tissue remained as a thin rim on periphery of the tumor; nevertheless it contained significant number of mitoses (Figure 6(d)) with their maximum at 28 day after hepatoma inoculation (Figure 4(c)). 

### 3.4. Blood and Peritoneal Cell Counts

**Figure 7:**
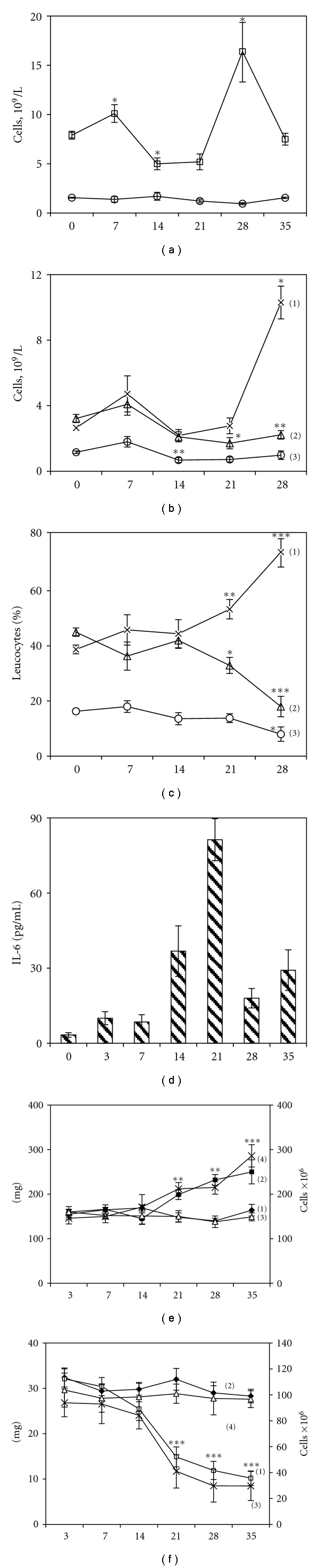
The influence of tumor growth on blood leukocytes, cytokine serum level, spleen, and thymus. (a)—blood leukocyte (upper curve) and peritoneal cell (lower curve) counts during the growth of hepatoma 22a. (b)—absolute cell numbers of leukocytes in the blood of tumor-bearing mice: polymorphonucleus leukocytes (1), lymphocytes (2), and monocytes (3). (c)—percent of total number of leukocytes: polymorphonucleus leukocytes (1), lymphocytes (2), and monocytes (3). Each point represents average values of 10 mice (M ± SEM), *P < .05, **P < .01, ***P < .001 compared to control. (d)—serum IL-6 level determined by ELISA, pg/mL. Each bar represents average values of 5 mice (M ± SEM). At every timepoint the data were compared to appropriate control group of mice. In (a)—(d) all data from control mice were combined into groups shown on day 0 (M ± SEM). (e)—spleen weight, mg (2: tumor-bearers, 3: control mice) and total number of nucleated cells per organ, mln (1: control mice, 4: tumor-bearers). (f)—thymus weight, mg (1: tumor-bearers, 2: control mice), and total number of nucleated cells per organ, mln (3: tumor-bearers, 4: control mice). Each point represents average values of 10 mice (M ± SEM).

The number of nucleated cells in the peritoneum was not significantly changed during the time of observation (Figure 7(a)) and averaged about 4 × 10^6^ cells/mouse. The cellular composition of PEC in experimental groups did not differ from the control groups and consisted approximately of 45–50% of macrophages, 50–55% of lymphocytes, and 5% of neutrophils and mast cells. 

Nevertheless, the growth of hepatoma influenced the amount and composition of peripheral white blood cells. Total leukocyte count was increased on day 7 and 28 (Figure 7(a)), which coincided with increased NO and ROI production in peritoneal macrophages. At 14 days we observed a decrease in total leukocyte number and diminished absolute monocyte count. Leukemoid reaction at the late stages of tumor growth was characterized mostly by progressively growing number of polymorphonuclear leukocytes (Figures 7(b) and 7(c)). However, the relative percentage and absolute number of blood monocytes were not significantly changed. On the contrary, from day 21 on lymphopenia was developed.

Control male mice of C3HA strain used in our experiments possessed about 45.0 ± 1.53% lymphocytes, 38.7 ± 1.6% polymorphonuclear cells, and 16.3 ± 0.9% monocytes of total number of peripheral blood leukocytes that differed from the Jackson laboratory Phenome database for male C3H/HeJ mice that have 68% lymphocytes, 32% polymorphonuclear cells (including 24% neutrophils, 4% eosinophils and 4% basophils), and 2% monocytes.

### 3.5. Thymus and Spleen

Since we observed significant changes in blood leukocyte counts, we were also interested to find some quantitative changes in spleen and thymus. Starting from the 21 day of hepatoma growth thymus weight was dramatically decreased which was also followed by significant reduction of the total number of thymocytes per organ (Figure 7(f)). At the same time we observed progressively growing splenomegaly (Figure 7(e)).

### 3.6. Cytokines in Blood and Tumor Supernatants

Serum levels of proinflammatory cytokine TNF-*α* and angiogenic factor VEGF did not differ from the control animals at 3, 7, 14, 21, 28, and 35 days of hepatoma growth (data not shown). The level of IL-6 was changed during tumor growth without any correlations to activation waves observed in peritoneal macrophages (Figure 7(d)). 

Neither TNF-*α* nor IL-6 was detected in supernatants of hepatoma 22a cells by ELISA. Therefore the elevation of IL-6 level in the circulation cannot be attributed to its production by tumor cells. However, hepatoma 22a was found to be a VEGF-producing tumor. During 48 h in culture tumor cells released up to 200 pg/mL VEGF as detected by ELISA. VEGF mRNA synthesis in these cells was also previously confirmed by RT-PCR [25].

## 4. Discussion

During the past decade, there appeared significant amount of new data indicating that dying cells produce variety of signals inducing innate immune responses [26]. It is now assumed that damage-associated molecular patterns (DAMPs) released from dead cells, like High Mobility Group Box 1 protein (HMGB1), heat shock proteins (HSP), ATP, IL-1*α*, hyaluronan fragments, and others can attract blood cells and induce sterile inflammation.

Spontaneous development of necrosis is an inherent property of solid tumors and is remarkably evident especially in fast-growing animal neoplasms. This phenomenon occurs due to inadequate blood supply, coincides with oxygen depletion, nutrient, and energy deprivation, and was studied in details [11]. From this point of view tumor tissue represents a combination of live and dead tumor tissues that may produce diverse signals to the host. These two parts of the tumor tissue are tightly interconnected and constantly influence each other. 

The notion that necrotic tumor cells could provide signals to enhance the growth of remaining viable ones has been considered over 50 years [27]. It has now become evident that hypoxia and the ensuing death of tumor cells are the most potent signals triggering neoangiogenesis in solid neoplasms [28]. The ensuing chronic hypoxia results in the generation of necrotic areas within the tumor, accompanied by the release of DAMPs. Hypoxia and DAMPs attract macrophages, which have potent proangiogenic properties. The cascade of underlying events sequentially involves the exponential growth of tumor cells, which overwhelms the functional capacity of the pre-existing vasculature and this gives start to a new wave of tumor death. Therefore, one can assume that tumor growth possesses a cyclical character with waves of tumor proliferation and cell death changing one another.

While producing many chemokines, live tumor cells have a strategy to recruit inflammatory cells from the circulation in order to have additional resource of growth factors and angiogenic activity [3]. Hypoxic, stressed, and dying tumor cells as well as extracellular matrix from necrotic areas represent a large panel of damage signals, being also chemoattractive and activatory for phagocytes [29]. Both types of these signals can reach distant sites of the body and influence immune cells.

Histological study of nonimmunogenic solid hepatoma 22a transplants revealed the appearance of first necrosis on day 14, and later on, this process developed progressively up to 35 day (the end of experiment). We may suppose that factors released from dead cells will be increased constantly in the circulation and will influence immune cells in a monotone manner. Unlike this, the behaviour of viable tumor tissue had a discrete character with periods of fast and slow growth. Histological examination discovered two dramatic time-points for the growth of murine hepatoma: the 7th and 28th days. 

Day 7 of the tumor growth was characterized by the appearance of first small blood vessels and mononuclear infiltration around the tumor. It is assumed that as soon as tumor nodule reaches the size beyond 2-3 mm, its development entirely depends on formation of a unique tumor vasculature to meet increased metabolic demands of fast growing malignant tissue [30]. We consider this period of tumor growth as so-called “angiogenic switch”. At this stage tumor nodule was small, contained increased number of mitoses, and had no any signs of necrosis. 

The 4th week of hepatoma growth was characterized by more moderate rate of growth but still at 28 day the volume of viable tumor tissue and rate of the tumor growth within this week were the largest during experiment. On day 28 we also observed maximal numbers of mitosis in live tumor tissue. Therefore, we suggested that both 7 and 28 days may be characterized by high angiogenic demands of the tumor. Later on, day 35 showed decreased mitotic activity, loss of viable tumor tissue, and dramatic increase in necrosis.

While summarizing all tumor-dependent effects, immunological changes during the growth of hepatoma fell into two categories: those that went in parallel steadily to the development of necrosis and others that differed significantly and showed pulsed dynamics. To the first type belonged thymic involution, lymphopenia, splenomegaly, and leukemoid reaction; to the second-changes in peripheral macrophage functional activity. 

The existence of a common mechanism for the development of the first group of changes is not proved. However, the coincidence of these alterations was first mentioned during the study of several mammary adenocarcinomas in mice [31]. Some carcinomas (CE carcinoma) induced changes similar to hepatoma 22a: thymic involution, lymphocyte depletion, granulocytosis, and splenomegaly, while others (1460 MCA tumor) induced neither of these changes. Later on, the association of thymic involution, splenomegaly, and myeloid reaction was confirmed during VEGF administration, and this factor was suggested to be a mediator of these processes [32, 33]. In our experiments, such changes occurred in parallel to the development of necrosis in hepatoma. Recent studies indicate the important role of IL-1*α* released from dead cells for the induction of granulocytosis [26].

Changes in the activity of peritoneal macrophages did not resemble the development of any of the abovementioned processes. In contrast to evident quantitative changes in the numbers of thymocytes and splenocytes as well as in blood granulocytes and lymphocytes, there was not any numerical difference in the number of peritoneal macrophages as well as in the number of blood monocytes during the whole experiment. Opposite to these we observed certain modifications of macrophage inflammatory phenotype every week. Several features, including NO_2_-production and 5′-N activity, changed up and down, that may possibly reflect the prevalence of different types of factors. One specific phenotype was repeated on 7 and 28 days of tumor growth and represented two peaks of macrophage activation when all inflammatory parameters were increased (Table 3). These changes of macrophage activity fit exactly to the time of described peaks of hepatoma activity.

Several changes on 7 and 28 days, like elevation of nitroxide as well as superoxide production (assessed by NBT-test), are known to be a feature of classically activated macrophages or M1 phenotype [2]. But there is some difficulty in attributing elevation of cell surface-associated enzyme 5′-N activity. 

Similar changes of ecto-enzyme activity in macrophages were also shown in mice bearing lymphomas [23] and in Syrian hamsters with highly malignant embryonic tumors [34] and were regarded as a sign of immunodepression. At the same time, others attribute increase of 5′-N to alternatively activated macrophages [24]. However, direct effect of Th2-type cytokines (IL-4, IL-13) on 5′-N expression has not yet been elucidated. Enhancement of 5′-N activity in vitro was documented quite rarely and was found in macrophages cultured in the presence of adenine nucleotides (ATP, ADP, AMP) [35]. Oppositely to this, decrease of 5′-N activity is a standard effect of macrophage activation in vitro. 5′-N quickly disappears from the plasma membrane of macrophages activated by LPS/IFN-*γ* and thus is considered to be a biochemical marker for classical type of macrophage activation [22]. 

Decrease in 5′-N activity in macrophages was also observed in our experiments on 3 and 14 days of hepatoma growth. But other parameters investigated at this period of time did not allow to attribute these activities to M1 type. Downregulation of NO_2_ production (3 and 14 days) and increased pinocytosis (14 day) are considered to characterize alternatively activated or M2 macrophages [36]. 

Independently of their interpretation these data allow to suggest that changes of 5′-N activity in tissue macrophages are a specific feature of tumor growth. CD73 (5′-N) is a GPI-linked cell-surface enzyme of purine metabolitic pathway which dephosphorylates AMP to adenosine [37]. Alteration of 5′-N activity in macrophages during tumor growth may reflect systemic changes in purine metabolism. It is also known that 5′-N activity can regulate leukocyte trafficing [37]. Other nonenzymatic functions of the CD73 molecule have also been suggested, for example, mediation of cell-cell and cell-matrix adhesions [38]. 

Taken together alterations in macrophage activity on days 3, 7, 14, and 28 could be characterized by “mixed” phenotype. The day 21 was characterized by almost no changes in macrophage functional activity with the exception for increased 5′-N activity.

At the late stage of tumor growth (day 35) we did not observe any signs of inflammatory response in macrophages. On the contrary, there was a decrease in superoxide anion production that can be considered as a sign of exhaustion after prolonged activation. Nevertheless, we cannot assume these changes as depression because they were followed by increase in VEGFR-1, VEGFR-2, and TSP-1 mRNA expression. These activities may be regarded as functions related to chemotaxis via VEGF receptors [39, 40] and phagocytosis of dead cells mediated by TSP-1 [41]. Expression of VEGFR-2 in peritoneal macrophages was also described in the literature in mice bearing orthotopic pancreatic tumors [42]. It is believed that macrophages from peripheral tissue do not recirculate; however, theoretically they can emigrate towards draining lymph nodes. 

Additionally we did not found any increase in the mRNA synthesis of proangiogenic cytokine VEGF in macrophages. Apart from this, we found, for the first time, increased expression of TSP-1 mRNA in peritoneal macrophages and thymocytes, the factor known to have antiangiogenic properties [13]. Elevation of TSP-1 may be connected with ATP release as one of the damage signals, shown to be an activator of TSP-1 secretion by monocyte-derived dendritic cells [43]. 

To conclude we found that the s.c. growth of nonimmunogenic hepatoma in mice induced in peripheral macrophages diverse forms of inflammatory responses. In available literature we found contradictory results concerning activity of macrophages isolated from sites distal to tumor. Different tumor models revealed specific changes in macrophage activity. In particular, in case of syngeneic fibrosarcoma or AK-5 tumor, NO-production by peritoneal macrophages was increased [44, 45] whereas in a model of growing mammary adenocarcinoma it was significantly decreased [46]. Interestingly, both positive and negative changes were equally interpreted as the signs of immunodepression: in the former case enhanced NO-production by macrophages was considered as a negative effect due to its suppressive influence on T-cell responses [44], whereas decreased NO-production reflected a lowered tumoricidal capacity of macrophages, thus representing a suppressed phenotype on its own [46]. In contrast to this, we did not observe significant immunodepressive activities in macrophages during hepatoma 22a growth. These variations can be ascribed to different tumor models. 

The most intriguing question was as follows: how did tumor cells activate distant macrophages? We suggested that at 7 and 28 days the macrophages could be activated by factors produced by live tumor cells and associated with angiogenic potential of the tumor cells. To elucidate this situation we examined the level of several cytokines in the blood of hepatoma-bearing mice throughout the time of experiment. For this study we selected angiogenic factor VEGF, chemotactic for blood monocytes [47], another well-known chemoattractant and activator of macrophages—TNF-*α* [48] and IL-6—a hepatocyte activating factor with indirect angiogenic activity [49]. Besides, IL-6 was recently shown to play a role in the induction of neutrophilia and downregulation of lymphopoiesis [50, 51].

Unfortunately, we did not find elevation of TNF-*α* in the circulation and it was not produced by hepatoma cells in vitro. Surprisingly, we similarly observed no enhancement of serum VEGF level in hepatoma-bearing mice, in spite of its production in vitro by cultivated cells. An explanation for this may be found in the different abilities of tumors to release VEGF into the circulation depending on the tumor structure, presence of capsule, and metastasis as it was shown for human hepatocelullar carcinomas [52]. Therefore we suppose that VEGF produced by hepatoma 22a may not necessarily enter the circulation but remain inside the tumor interacting with extracellular matrix. Oppositely to TNF-*α* and VEGF, serum level of IL-6 was changed in tumor-bearing mice but it did not correlate with alterations in macrophage activity or other effects.

We can also suggest that macrophage activity may be modulated by the molecules released from dying tumor. In particular, abundant tumor cell-derived substances like hyaluronan fragments together with heat shock proteins are endogenous ligands to TLR2 and TLR4 and trigger M2-like cytokine profile in macrophages [53]. Alternatively, HSP60 and HSP70 cause significant increase in NO production by peritoneal macrophages, thus shifting their activity to M1-phenotype [54, 55]. In addition, small hyaluronan fragments are also known to upregulate NO-production in macrophages [56] and ROI in human monocytes, thus contributing to M1-phenotype [57]. At the same time large hyaluronan molecules possess anti-inflammatory activity [58]. 

An important role in modulation of macrophage behaviour may also play purine metabolites. When any cell dies and loses membrane integrity, nucleotides and metabolites, such as ATP, adenosine, and uric acid, will be released to potentially alert the immune system, not only activating but also suppressing mononuclear phagocytes. Thus, ATP released from dying cells is known to activate macrophages and potentiate their superoxide generation [59] and pinocytosis [60]. Meanwhile, adenosine, appeared as a result of ATP/ADP breakdown via sequential ecto-nucleotidase reactions, is known to downregulate macrophage activity while inhibiting NO and ROI production [61]. 

The elevation of 5′-N activity in macrophages, observed in our experiments, presumably, may lead to increased concentration of extracellular adenosine and serve as an autoregulatory mechanism. On one hand, enhanced concentration of adenosine may dampen inflammatory reactions; on the other hand, intracellular adenosine after entering the cell becomes an important source of superoxide radical production via desamination process in activated macrophages [62].

In summary, our studies demonstrate that nonimmunogenic syngeneic tumor affects functional activity of distant macrophages, this influence being mostly of stimulatory character. We propose that peritoneal macrophages can receive two types of immunomodulatory signals produced by either live or dead tumor cells. Peritoneal macrophages do not directly participate in the dramatic events occurring at the tumor site. According to the modern knowledge these cells can only play a role of bystanders but their type of reaction reflects systemic immune response to tumor growth as a whole.

## Figures and Tables

**Table 1:** Results of the transplantation test.

Vaccinations	Challenge with live hepatoma cells			
	10^3^	10^4^	10^5^	10^6^
Cotrol (no vaccination):				
(i) Number of tumor takes	1/5	2/5	5/5	5/5
(ii) Mean tumor volume, cm^3^	0.01	0.29 ± 0.12	2.21 ± 1.20	7.66 ± 0.94
10^7^ irradiated hepatoma cells i.p.:				
(i) Number of tumor takes	1/5	3/5	5/5	5/5
(ii) Mean tumor volume, cm^3^	0.03	0.04 ± 0.01	3.10 ± 1.4	9.40 ± 2.60

Number of tumor takes (numerator), number of animals per group (denominator).

Tumor volume was measured on 28 day of tumor growth (M ± SEM). Control mice received 0,5 mL of PBS i.p.

**Table 2:** Rate of tumor growth per week.

Part of tumor tissue	Increase in the tumor tissue per week, cm^3^			
	7 day–14 day	14 day–21 day	21 day–28 day	28 day–35 day

Necrotic	0.01 ± 0.005	0.10 ± 0.02	1.20 ± 0.30	1.59 ± 0.35
Viable	0.29 ± 0.06	0.65 ± 0.11	0.73 ± 0.15	−0.34 ± 0.10

Tumor volume (M ± SEM) within the previous week (e.g., day 7) was subtracted from the tumor volume within the next week (e.g., day 14). Volume of viable tumor tissue within the last week decreased.

**Table 3:** Changes in macrophage activity during the growth of hepatoma 22a.

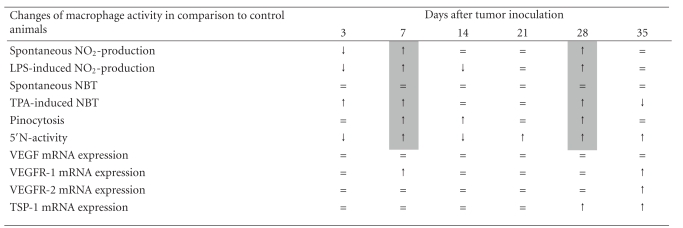

↑ increased parameter, ↓ decreased, or = no difference in tumor-bearing mice as compared to appropriate control.
